# Classification of arm swing as a clinical marker of advancing spinal deformity among community-dwelling female volunteers 60 years or older

**DOI:** 10.1038/s41598-019-43732-3

**Published:** 2019-05-20

**Authors:** Tetsuya Kobayashi, Shizuo Jimbo, Issei Senoo, Mutsuya Shimizu, Hiroshi Ito, P. T. Hisashi Chiba

**Affiliations:** 1 Department of Orthopaedic Surgery, Asahikawa Medical University, Asahikawa, Japan; 2 Department of Rehabilitation, Furano Kyokai Hospital, Furano, Japan

**Keywords:** Physical examination, Epidemiology

## Abstract

The clinical characteristics of adult spinal deformity (ASD) include worsening of deformity during gait, which leads to unstable posture and propensity to fall. The purpose of this study was to classify arm swing and to analyse its clinical implications. Clinical and radiographic evaluations were performed with 168 community-dwelling female volunteers recruited from a population register in Hokkaido, Japan, with a mean age of 67.3 ± 4.7 years, and arm swing was classified into four groups according to maximum forward and backward arm swing distance: (1) predominantly forward swing with forward swing always larger than backward swing (FS, n = 138), (2) equal or equivocal swing (ES, n = 8), (3) predominantly backward swing with backward swing always larger than forward swing (BS, n = 20), and (4) thigh-hand type without arm swing with their hands placed on thighs (TH, n = 2). BS and FS showed significant differences in radiographic lumbar lordosis (BS 19.4 ± 18.1° vs. FS 40.6 ± 14.5°, P < 0.01 ANOVA), pelvic tilt (BS 40.0 ± 7.3° vs. FS 22.9 ± 8.9°, p < 0.01), number of vertebral fractures (BS 1.2 ± 1.4 vs. FS 0.3 ± 0.6, p < 0.01), and trunk extensor muscle strength (BS 374.9 ± 134.8 N vs. FS 478.1 ± 172.6 N, p < 0.05). Arm swing correlated with severity of radiographic ASD, osteoporotic changes, and back muscle weakness. The number of ASD patients, which includes patients with *de novo*/idiopathic scoliosis, degenerative/osteoporotic kyphosis, and other neuromuscular deformities, has been increasing, and further study should clarify the importance of dynamic evaluation of ASD among elderly patients.

## Introduction

Increasing activity levels among the elderly population have shed light on the problems of adult spinal deformity (ASD), which include *de novo*/idiopathic scoliosis, degenerative/osteoporotic kyphosis, and other neuromuscular deformities, and our 12-year longitudinal study revealed a decrease in lumbar lordosis predominant among elderly patients^1^. Recent studies of ASD have revealed that spinal deformity is related to pelvic anatomy and changes in pelvic rotatory position^1,2^. Spinopelvic alignment has been widely used to evaluate ASD, and in 2012, Schwab and the Scoliosis Research Society proposed the SRS-Schwab ASD classification including pelvic incidence and pelvic tilt^2^. A decrease in lumbar lordosis is associated with posterior rotation of the pelvis, hip extension, and knee flexion, all of which compensate for spinal deformity to balance the upright posture. ASD patients show worsening of the kyphotic deformity during gait due to precluded pelvis-hip-knee compensation and back muscle weakness^3^. To date, ASD studies have focused on the static deformity using upright entire spine radiographs^2,4^, and modalities to evaluate dynamic ambulatory kyphosis are scarce. We reported changes in trunk angle using digitally captured gait postures^5^ and found some characteristic changes in arm swing among elderly participants that had not been previously elaborated.

The purpose of this study was to evaluate gait posture using arm swing and to analyse its clinical implications.

### Methods

**Figure 1:**
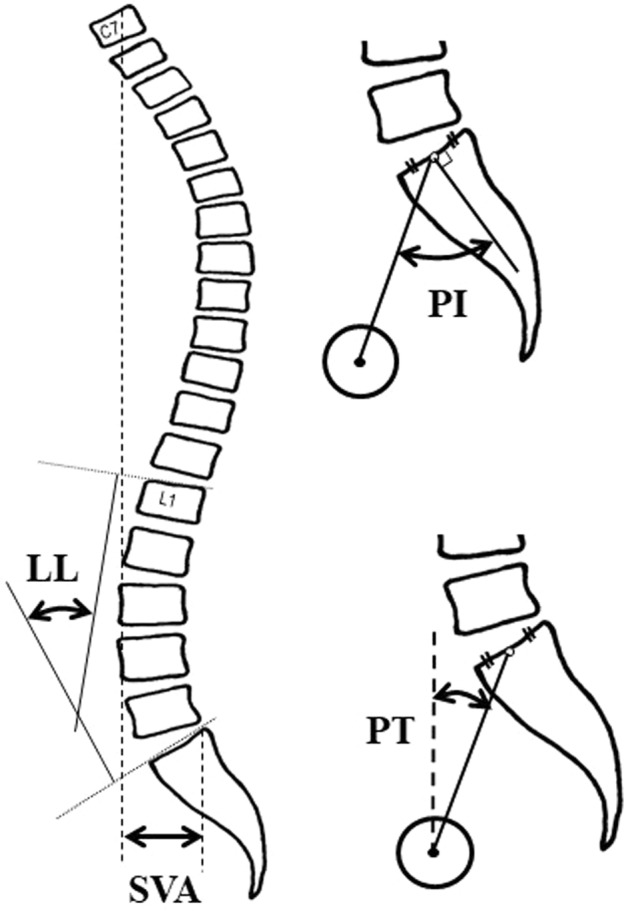
Standardized radiographic measurements. LL is the angle between the upper endplate of L1 and S1. SVA is the distance between plumb lines through the center of C7 vertebral body and the posterosuperior corner of S1. PI is the angle between the line through center of femoral head and the midpoint of the sacral table and the line perpendicular to the sacral table. PT is the angle between the line through the center of the femoral head and the midpoint of the sacral table and the vertical reference.

Among 202 female volunteers who were recruited from the population register in Hokkaido, Japan, 168 participants were included according to the following inclusion criteria: (1) 60 years or older, (2) walking independently without aid, (3) having provided a written informed consent. Upright lateral entire spine radiographs were taken and used for standardized radiographic measurements including lumbar lordosis (LL), pelvic tilt (PT), sagittal vertical axis (SVA), and pelvic incidence (PI) (Fig. 1).

The sum of the sagittal modifiers of the SRS-Schwab ASD classification (difference in PI and LL angles or PI-LL mismatch, PI-LL 0 for <10°, 1 for 10–20°, 2 for >20°; PT 0 for <20°, 1 for 20–30°, 2 for >30°; SVA 0 for <4 cm, 1 for 4–9.5 cm, 2 for >9.5 cm; Schwab-SM) was used to evaluate the severity of ASD and was applied to both idiopathic and degenerative deformites^2^. The number of vertebral fractures (VF) was evaluated using quantitative and semiquantitative (SQ) methods; more than a 25% decrease in anterior vertebral height, more than a 20% decrease in middle vertebral height compared to posterior vertebral height, or more than a 20% decrease in any vertebral height compared to adjacent vertebrae between T4 and L5^6^, along with a vertebral body with SQ grade 2 or 3^7^, was defined as VF. Physical measurements included: range of motion (ROM) of active lumbar extension (distance from floor to the sternal notch at maximal active back extension in the prone position with fixed legs, BET), ROM of passive knee extension (KEX), and isometric trunk extensor muscle strength (EX). Trunk muscle strength was measured using a chair-type isometric dynamometer (GT350, OG Giken Co., Japan). Adjustable straps were attached to fix the examinee’s trunk, pelvis, and thighs to the device. The examinee was then instructed to perform maximal isometric trunk extension until peak-out, and the best results were used after three trials. They were also monitored for their back symptoms and instructed to stop the trial when it became painful.

Trunk inclination angle (TIA) was recorded as the angle between a line connecting surface markers attached on the C7 (on the most prominent cervical vertebra) and L4 spinous processes (on the intercrestal line) and the vertical reference line. The difference of TIA between walking and standing at rest was calculated (dTIA = TIA walking − TIA at rest), with large values indicating increased ambulatory kyphosis^5^.

**Figure 2:**
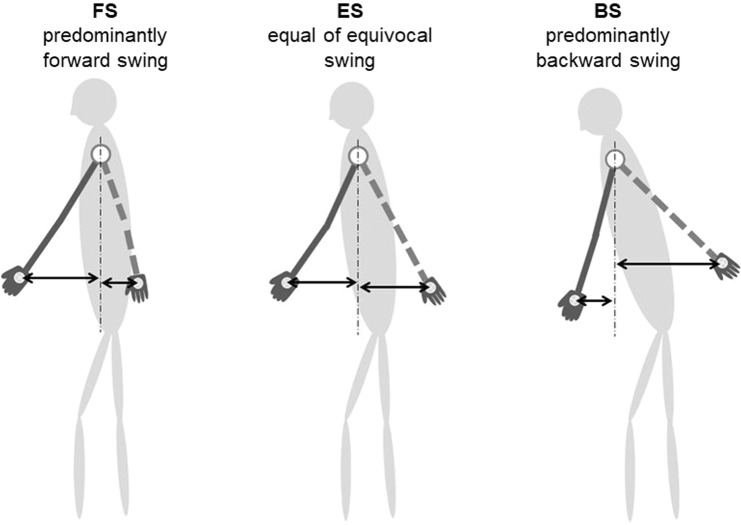
Classification of arm swing type. Arm swing was classified using the distance between plumb lines through center of the shoulder joint and the center of the palm at the terminal stage of the recorded forward and backward arm swing; FS type is defined as forward swing always larger than backward swing, ES type is defined as equal or equivocal swing, and BS type is defined as backward swing always larger than forward swing.

**Figure 3:**
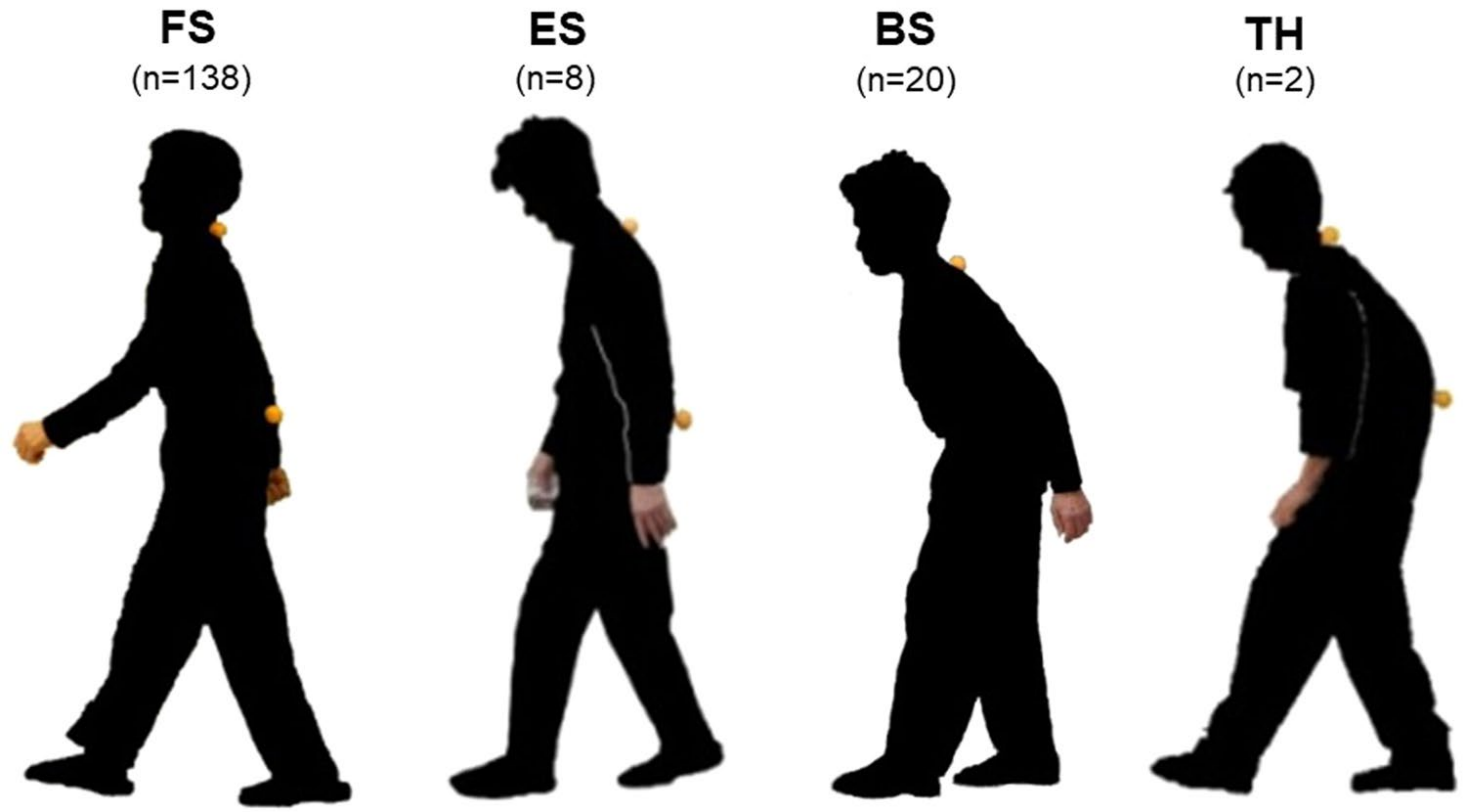
Images of arm swing type among 168 community-dwelling volunteers. There were two participants without arm swing during gait, with their hands placed onto their thighs; classified as TH type.

Participants were instructed to stand at the start line of a 6-meter walkway and to look straight forward with a relaxed posture. Then, they were instructed to walk freely at a comfortable speed along a straight line on the floor, without specifying their body posture or arm swing. Walking trials were repeated at least twice until the attending examiner could observe and record reliable gait posture. Gait posture was captured using a commercially available digital camcorder fixed at 1 meter from the floor and 6 meters from the walkway. Arm swing type was classified into four groups according to maximum forward and backward arm swing distances measured between the plumb lines through the centre of the shoulder joint and the centre of the palm at terminal swing; (1) predominantly forward swing type (FS) with forward swing always larger than backward swing, (2) equal or equivocal swing type (ES), (3) predominantly backward swing type (BS) with backward swing always larger than forward swing, or (4) thigh-hand type (TH) without arm swing with their hands placed on the thighs (Figs 2, 3). To clarify the clinical characteristics of participants with ambulatory kyphosis, subgroups with increased dTIA, defined as dTIA ≥ 8.0°, were selected and their clinical and radiographic parameters were compared according to their arm swing types.

Physical measurements were performed by experienced physical therapists according to the instructions of Japanese Orthopaedic Association, which have been widely adopted in the rehabilitation settings, and the details of our physical measurement and measurement errors have been reported^8^.

For continuous variables, statistical significance was determined using analysis of variance (ANOVA) or independent t-test, as appropriate. If a significant difference was observed by ANOVA, a pairwise comparison was performed using Fisher’s test of least significant difference (LSD). Since we had a small sample size in some subgroups, we checked and verified the assumptions prior to the application of parametric methods for statistical significance. In the current study, TH group (n = 2) was excluded from statistical analyses.

This study was a component of our ongoing cohort study, Asahikawa Observational Study of Spinal Aging in a Prospective Cohort (the ASAP study), which has been recruiting adult volunteers from a population register since 1983. This study basically aimed to analyse the epidemiological aspects of degenerative spinal deformities in designated farming districts in Hokkaido, Japan^3^. Current participants should be representative of the whole female population in their respective age groups, however, considering our inclusion criterion of independent walking, they might represent a healthier subgroup. Asahikawa Medical University Ethical Review Board approved the utilization and publication of the study data, and all methods were performed in accordance with the relevant guidelines and regulations^4–6^. All participants submitted written informed consent for study participation. The datasets generated during and/or analysed during the current study are available from the corresponding author on reasonable request.

## Results

The demographics of the 168 participants were as follows: age 67.3 ± 4.7 years, body height 150.3 ± 6.4 cm, body weight 56.3 ± 8.8 kg, LL 37.3 ± 16.7°, PT 25.4 ± 10.9°, SVA 2.2 ± 3.7 cm, PI 54.1 ± 10.3°, BET 10.5 ± 5.7 cm, EX 462.8 ± 174.8 N, KEX −0.9 ± 6.2°, and dTIA 4.7 ± 3.7°. Arm swing classification revealed that FS type was most frequent (n = 138) followed by BS (n = 20), ES (n = 8) and TH (n = 2), and the measured variables in each arm swing type are shown in Table 1.

**Table 1:** Arm swing type and clinical characteristics among 168 female volunteers (^†^p < 0.05; ^‡^p < 0.01 ANOVA LSD).

	FS	ES	BS	TH
n	138	8	20	2
Age (years)	67.0 ± 4.7	66.9 ± 3.1	68.6 ± 5.3	71.5 ± 5.0
dTIA (degree)	4.0 ± 3.0^**‡**^	3.9 ± 1.3	9.0 ± 4.9^**‡**^	11.9 ± 1.0
LL (degree)	40.6 ± 14.5^**†, ‡**^	29.0 ± 20.1^**†**^	19.4 ± 18.1^**‡**^	20.5 ± 2.1
PT (degree)	22.9 ± 8.9^**‡**^	28.6 ± 13.2^**†**^	40.0 ± 7.3^**†, ‡**^	52.5 ± 14.8
Schwab-SM	1.8 ± 1.6^**‡**^	2.8 ± 2.3^**†**^	4.5 ± 1.2^**†, ‡**^	6.0 ± 0.0
VF (number)	0.3 ± 0.6^**‡**^	0.4 ± 0.5^**†**^	1.2 ± 1.4^**†, ‡**^	2.0 ± 1.4
BET (cm)	11.6 ± 5.3^**‡**^	9.1 ± 4.7^**†**^	3.6 ± 3.4^**†, ‡**^	—
EX (N)	478.1 ± 172.6^**†**^	465.1 ± 221.0	374.9 ± 134.8^**†**^	—
KEX (degree)	−0.3 ± 6.2	−1.3 ± 2.5	−3.8 ± 5.8	—

Subjects with TH could not provide reliable physical measurements for BET, EX, and KEX. FS- forward swing, ES- equal or equivocal swing, BS- backward swing, TH-thigh-hand type, dTIA- difference of trunk inclination angle, LL- lumbar lordosis, PT- pelvic tilt, Schwab-SM- Schwab sagittal modifier, VF- vertebral fracture, BET- back extension test, EX- trunk extensor muscle strength, KEX- knee extension angle.

Radiographic ASD parameters deteriorated from FS to ES, BS, and TH in order: LL FS 40.6 ± 14.5°^**†‡**^, ES 29.0 ± 20.1°^**†**^, BS 19.4 ± 18.1°^**‡**^, TH 20.5 ± 2.1°; PT FS 22.9 ± 8.9°^**‡**^, ES 28.6 ± 13.2°^**†**^, BS 40.0 ± 7.3°^**†‡**^, TH 52.5 ± 2.1°; Schwab-SM FS 1.8 ± 1.6^**‡**^, ES 2.8 ± 2.3^**†**^, BS 4.5 ± 1.2^**†‡**^, TH 6.0 ± 0.0, VF FS 0.3 ± 0.6^**‡**^, ES 0.4 ± 0.5^**†**^, BS 1.2 ± 1.4^**†‡**^, TH 2.0 ± 1.4 (^**†**^p < 0.05; ^**‡**^p < 0.01 ANOVA LSD).

A comparison of physical parameters showed that dTIA was significantly larger in BS than in FS (BS 9.0 ± 4.9°, FS 4.0 ± 3.0°, p < 0.01 ANOVA LSD), and EX was significantly weaker in BS than in FS (BS 374.9 ± 134.8 N, FS 480.0 ± 174.7 N, p < 0.05 ANOVA LSD).

Among participants with increased ambulatory kyphosis, defined as dTIA ≥ 8.0°, kypho-BS (n = 20) and kypho-FS (n = 10), independent t-test showed significant differences in LL (kypho-BS 19.4 ± 18.1°, kypho-FS 45.8 ± 20.9°, p < 0.01), PT (kypho-BS 40.0 ± 7.3°, kypho-FS 27.8 ± 10.4°, p < 0.01), Schwab-SM (kypho-BS 4.5 ± 1.2, kypho-FS 2.3 ± 1.9, p < 0.01), VF (kypho-BS 1.2 ± 1.4, kypho-FS 0.4 ± 1.0, p < 0.05), BET (kypho-BS 3.6 ± 3.4 cm, kypho-FS 10.2 ± 9.0 cm, p < 0.01), and KEX (kypho-BS −3.8 ± 5.8°, kypho-FS 2.5 ± 2.9°, p < 0.01) (Table 2).

**Table 2:** Arm swing type and clinical characteristics among participants with increased ambulatory kyphosis (dTIA ≥ 8°).

	Kypho-FS	Kypho-BS	p-value (Independent t-test)
n	10	20	
Age (years)	66.6 ± 5.8	68.6 ± 5.3	n.s.
dTIA (degree)	10.5 ± 1.9	9.0 ± 4.9	n.s.
LL (degree)	45.8 ± 20.9	19.4 ± 18.1	<0.01
PT (degree)	27.8 ± 10.4	40.0 ± 7.3	<0.01
Schwab-SM	2.3 ± 1.9	4.5 ± 1.2	<0.01
VF (number)	0.4 ± 1.0	1.2 ± 1.4	<0.05
BET (cm)	10.2 ± 9.0	3.6 ± 3.4	<0.01
KEX (degree)	2.5 ± 2.9	−3.8 ± 5.8	<0.01

Kypho-FS- forward swing with dTIA ≥ 8°, Kypho-BS- backward swing with dTIA ≥ 8°, dTIA- difference of trunk inclination angle, LL- lumbar lordosis, PT- pelvic tilt, Schwab-SM- Schwab sagittal modifier, VF- vertebral fracture, BET- back extension test, KEX- knee extension angle, n.s.- not significant.

## Discussion

The current study showed that BS type arm swing was associated with decreased LL, increased PT, knee flexion contracture, decreased lumbar ROM with back muscle weakness, and increased number of vertebral fractures, all of which indicated advanced degenerative spinal deformity.

Age-related changes in gait pattern in the elderly have been documented, and common findings include decreased gait speed and impaired balance control^9^. Gait speed has been evaluated as an important marker of physical function, and decreased gait speed has been used to diagnose frailty and persons with sarcopenia^10,11^.

Ambulatory kyphosis, measured by dTIA in the current study, has been reported as one of the characteristic features of age-related spinal deformity^3,5^. Merchant *et al*. indicated that ambulatory kyphosis was a better marker than gait speed in predicting decline in function and subsequent frailty^12^. They studied 90 community-dwelling participants between the ages of 60 and 80 years, and postural adaptation with increased trunk stooping angle preceded the decline in gait speed. They concluded that trunk posture adaptation, before the onset of gait slowness, should be included for planning interventions for sarcopenia and frailty.

Arm swing is usually neglected in modern-day gait analyses^13,14^, and no study has reported the relationship between arm swing and degenerative spinal deformity.

Wolfson *et al*. used inconsistency, arrhythmicity, and amplitude in arm movement to introduce a gait abnormality rating scale (GARS) and reported that a higher GARS score was associated with fall risk after studying 49 nursing home residents^15^.

It has been observed that elderly persons tend to walk with decreased arm swing amplitude, and this has been indicated as the earliest marker of age-associated basal ganglia dysfunction^16^. Mirelman *et al*. mentioned that the pendulum-like motions of the arms act as passive dampers and improve stability and the energy efficiency of aged gait, and their study showed that increased arm swing was associated with increased gait speed^17^.

Our study showed that dTIA and ASD parameters became worse from FS to TH, which suggested FS as the standard form of arm swing, ES as aged type with decreased arm swing amplitude, BS as characteristic of ASD with increased ambulatory kyphosis with counter-balancing arm swing direction, and TH as the final form of independent gait. In the current study of community-dwelling volunteers, the most advanced TH type was too small in number to provide statistical significance. Further study of ASD patients should provide different prevalence of arm swing types and enough statistical power to clarify clinically important differences for the ASD parameters used.

Even among the participants with increased ambulatory kyphosis, defined as dTIA ≥ 8.0°, FS was associated with better clinical and radiographic conditions than BS, and BS showed characteristics of advanced radiographic ASD, osteoporotic changes, and joint contractures. Arm swing modification from BS to FS after conservative or surgical treatment of ASD might indicate postural or gait adaptation.

The clinical importance of arm swing evaluation indicated that it was a simple measure possible without using special devices in daily practice yet provided a relevant estimation of the severity of age-related conditions including ASD, osteoporosis, and muscle weakness associated with BS type arm swing.

Limitations include that the current study included only female participants with relatively active and healthy backgrounds, and despite observing some statistical significance in the larger subgroups, the number of participants was too small in BS, ES, and TH subgroups to reach definite conclusions. Future studies should aim to determine what the clinical significance of certain degrees of anterior or posterior arm swing amplitudes are, as well as test-retest repeatability of arm swing subgrouping classification. In the current study, radiographic ASD criteria could not discriminate mixed diagnosis such as idiopathic, *de novo*, degenerative, and neuromuscular deformities, and further study of these separate diagnoses should clarify detailed clinical characteristics, with different prevalence of arm swing types. The clinical relevance of arm swing in males should be studied in a larger cohort. Radiographic measurements were performed in a standardized manner; however, physical evaluations, performed by designated physical therapists and adopted from commonly used methods in the rehabilitation settings, involve inherent error and the number of participants should be large enough to reach significant results. Our previous studies validated the aforementioned methods^5,8,18^.

The proposed classification of arm swing should be mainly for diagnostic purposes, and arm swing evaluation should be a simple and easy examination in daily practice. This study justifies more study of dynamic gait effects on spinal deformities. Advances in dynamic monitoring devices might add useful diagnostic and outcome measures of ambulatory kyphosis. Further studies should clarify the clinical importance of arm swing and other dynamic variables for evaluating the ever-increasing population of elderly patients with spinal deformity.
